# Improving Collaborative Behaviour Planning in Adult Auditory Rehabilitation: Development of the I-PLAN Intervention Using the Behaviour Change Wheel

**DOI:** 10.1007/s12160-016-9843-3

**Published:** 2017-12-12

**Authors:** Fiona Barker, Simon de Lusignan, Cooke Deborah

**Affiliations:** 1 Department of Clinical Medicine and Aging, University of Surrey, Guildford, UK; 2 School of Health Sciences, University of Surrey, Guildford, UK

**Keywords:** Audiology, Behaviour change, Behavioural change wheel, Intervention development

## Abstract

**Background:**

The consequences of poorly managed hearing loss can be ameliorated with hearing aid use but rates of use are sub-optimal. The impact of audiologist behaviour on subsequent use, particularly over the long term, is unknown.

**Purpose:**

This study aimed to describe the role of the behaviour change wheel in developing an intervention to introduce and embed particular clinical behaviours into adult hearing aid fitting consultations, within the framework of the Medical Research Council guidance on complex interventions.

**Methods:**

Following the steps of the behaviour change wheel, audiologist behaviours that might influence hearing aid use were identified based on a systematic review and qualitative work with audiologists. An analysis, using the COM-B model, identified potential drivers of the target behaviours. This was used to select intervention functions and behaviour change techniques likely to influence behaviour in this context.

**Results:**

The target behaviours were as follows: giving information about the benefits of hearing aid use and the negative consequences of non-use, providing prompts for use and engaging in collaborative behavioural planning for use. The behavioural analysis suggested that psychological capability, opportunity and motivation were potential drivers of these behaviours. The intervention functions of education, coercion, training, environmental restructuring, modelling and enablement were selected and combined to develop a single complex intervention that seeks to address the target behaviours.

## Teaser

A description of how the Behaviour Change Wheel has been used to develop an intervention to introduce and embed selected behaviours in the clinical routine of audiologists.

## Background

Figures from the World Health Organisation suggest that hearing loss is the second most frequent sensory deficit affecting an estimated 360 million people worldwide [1, 2]. Hearing loss consistently ranks in the top 20 causes (out of 259 causes) of years lived with a disability [3]. The prevalence of hearing loss increases with age, which has serious implications for a global population in which the proportion of elderly people is rising at unprecedented rates according to the World Health Organisation [4]. The WHO estimates that approximately one third of the population aged over 65 have a disabling hearing loss [1, 2]. The standard management for adult onset hearing loss, at least in the developed world, is hearing aid fitting [5]. Most people fitted with hearing aids have autonomy over whether they are used or not, and research suggests up to 40 % of people who are fitted with hearing aids do not wear them [6–13]. In addition to the negative impacts on patients such as depression, cognitive decline and reduced quality of life [14–25], this represents an inefficient use of audiologists’ time and a considerable waste of resources especially in health economies where hearing aids are provided by the state. Previous reviews have explored the range of reasons for non-use [26, 27]. A strength of these reviews is that they have been informed by patient self-reported reasons for non-use such as difficulty manipulating the controls, poor perceived benefit, lack of social support or stigma. However, a potential limitation is that they have lacked a theoretical basis to structure the investigation and analysis of reasons for non-use. This means that some potential drivers of behaviour, particularly those where people may lack insight such as automatic or habitual behaviours, have been neglected as avenues for intervention development. Behavioural problems like this are not unique to hearing healthcare. It is estimated that between a quarter and a half of patients with chronic disease have problems adopting and maintaining behaviour such as taking medication and following a diet or exercise plan [28, 29].

A theory-based analysis of the factors that might influence hearing aid use suggests that audiologist behaviour, such as self-management support (SMS), might play an important role in influencing patient behaviour [30–34]. Some types of SMS can improve some outcomes for adults with hearing loss in the short term[35]. However, there is a lack of evidence regarding the effects of SMS that actively involves people with hearing loss in planning their own care, despite evidence from other long-term conditions that collaborative planning including goal-setting, action-planning and problem-solving helps patients change their behaviour and improve their health [36, 37]. There is also a lack of evidence for the long-term effects of any intervention including SMS—a serious issue in the context of a long-term condition [38, 39]. There is evidence suggesting further opportunities to use SMS in the context of audiology such as involving the patient and significant others in shared consideration of need and decision-making [40–43]. However, little research into the use of SMS has been undertaken at the hearing aid fitting stage of the patient journey [44] and no research has examined the role of behavioural theory in encouraging the routine implementation of SMS in this context.

The multi-component nature of SMS and the interrelationship of clinician and patient behaviour in the context of a long-term condition imply that any intervention targeting behaviour in this context will be complex. This research uses the Medical Research Council (MRC) model for the development and evaluation of complex interventions [45, 46]. The framework calls for the use of theory during the development phase of intervention development but provides no information or advice on how to choose an appropriate theory. Unfortunately, there is a confusing array of theories on offer, often with differently named but similar constructs. This has been cited as a reason why theory has been so rarely used and reported in studies seeking to change patient or healthcare professional behaviour [47–50]. In addition, there has been little guidance on moving from theory to intervention development [51].

Recently, a supra-theory model has been developed as a starting point for intervention development. Michie et al. propose that people need the capability (C), opportunity (O) and motivation (M) to perform a behaviour (B) and developed the COM-B model to guide understanding of behaviour in context and develop behavioural targets as a basis for intervention design [52]. The model provides a starting point and can signpost to psychological theories of, for example, motivation if a more granular theoretical understanding of behaviour is required. Importantly, the COM-B model has been developed as part of a larger system called the behaviour change wheel (BCW) [51, 52], which is designed to help intervention developers move from a behavioural analysis of the problem to intervention design in a systematic way using the evidence base. The steps of the BCW have been mapped onto the phases of the MRC framework by researchers seeking to integrate the use of theory with intervention design [53].

The BCW has been applied successfully in a number of contexts [54, 55]. It has been recognised as a potentially effective strategy to guide intervention development and implementation in audiological practice [56], but it has not yet been applied in this context. In this paper, we describe how each of the steps of the BCW has been operationalised to guide the development of an intervention in audiology. The intervention seeks to introduce and embed selected self-management support behaviours into the clinical routine of audiologists with the aim of improving the long-term use of hearing aids by adults with acquired hearing loss.

## Methods

**Fig 1:**
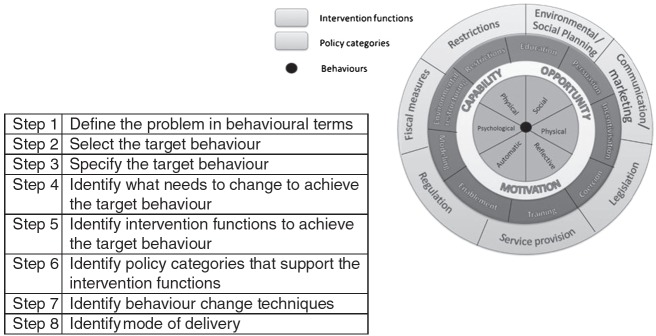
The steps of the BCW process with the COM-B model at the centre, intervention functions and policy categories

Using the integrated model of the MRC framework and BCW steps described by Sinnott et al. [53], we followed the BCW process [52] through the development phase of the MRC framework. Figure 1 describes the steps of the BCW process.

### Step 1—Define the Problem in Behavioural Terms

The distinctions and links between behaviour and outcome are poorly defined in the context of hearing loss [57]. We first searched the literature detailing the negative consequences of hearing loss for adults with acquired hearing loss and the literature detailing a link between hearing aid use (behaviour) and amelioration of these negative consequences (outcomes). This allowed us to define the problem of poor outcomes for people with acquired hearing loss in behavioural terms (low levels of hearing aid use).

We also searched the relevant literature on hearing aid use to establish the extent of and reported reasons for non-use. We identified several individual studies reporting rates of hearing aid use and non-use [6–13] and two recent systematic reviews that summarise reported reasons for non-use [26, 27]. We used the COM-B model to categorise reasons for non-use reported in these reviews.

### Step 2—Select the Target Behaviour

The second step of the BCW process is to select a target behaviour which could address the behavioural problem. All behaviours, including hearing aid use, occur within a system of other competing or contributing behaviours. To identify a target behaviour, we first carried out a systematic review of interventions to improve hearing aid use, using the Chronic Care Model [31] as a framework for our analysis. We were interested in the effect of the behaviour of providers on patient behaviour. The method for this systematic review has been published elsewhere [35].

To refine our target, we carried out a Delphi review of SMS in audiology [58] involving 26 stakeholders including hearing aid users, providers and researchers. The aim was to assess consensus on which key behaviours support self-management in this context. We then combined the results of the existing systematic reviews on hearing aid use, our own systematic review and the Delphi review to develop a conceptual map of behaviours relevant to hearing aid use. The behavioural target(s) were further refined during qualitative work with audiologists and patients to identify which of the potential candidate behaviours were already taking place and which were missing from routine hearing aid fitting consultations [59]. This information was used to inform our judgements on final target behaviour selection using the criteria set out in the BCW guide: the likely impact of changing the behaviour; the likelihood that behavioural change would be implemented, the spill-over or knock-on effect of change on other behaviours and the ease with which each behaviour could be measured [52].

### Step 3—Specify the Target Behaviour

In the third step, we developed a specification of who would perform the target behaviours, what they would need to do differently to achieve change, where and when they need to do it and, if necessary, how often and with whom.

### Step 4—Identify What Needs to Change

Moving from the MRC sub-phase of identifying the evidence base to identifying and developing theory, we used the COMB model as a framework for an analysis of potential drivers of the target behaviours, using semi-structured interviews. The methods for this qualitative analysis are published elsewhere [60].

### Steps 5 and 6—Identify Intervention Functions and Policy Categories

Using decision grids published in the BCW guide and the APEASE criteria [52], we selected intervention functions and policy categories to address the driving factors identified in the behavioural analysis (steps 5 and 6). The decision grids provide evidence- and theory-based guidance on which intervention functions and policy categories might be used to address particular COM-B elements. The APEASE criteria allow researchers to select context-appropriate intervention functions and policy categories based on affordability, practicability, effectiveness/cost-effectiveness, acceptability, side effects/ safety and equity.

### Step 7—Identify Behaviour Change Techniques

During the final stages of intervention development, we stepped outside the BCW to select specific behaviour change techniques to deliver the intervention functions selected in step 5. The BCW has been linked to a taxonomy of behaviour change techniques—the behaviour change technique taxonomy, version 1 [52, 61]. The taxonomy allows systematic and transparent selection of specific techniques that have been shown in practice or in theory to serve particular intervention functions. This allows the detailed components or active ingredients of an intervention to be linked back to theory via the BCW and COM-B analysis. Once again, the APEASE criteria were used to select individual behaviour change techniques to address each intervention function.

### Step 8—Identify Mode of Delivery

In the final step, we identified delivery options for the behaviour change techniques, giving careful consideration to the context in which this intervention will be implemented. Useful information was obtained from the audiologists who participated in the qualitative studies and this was supplemented by one of the researchers (FB) who has experience in audiological practice as a clinical scientist. This informed application of the APEASE criteria to select specific modes of delivery.

## Results

### Step 1—Define the Problem in Behavioural Terms

Our literature search suggested that many negative health and psychosocial consequences of poorly managed acquired hearing loss can be ameliorated by hearing aid use [20, 62, 63], providing a rationale for addressing hearing aid use. Two behavioural problems arose from this analysis: low levels of uptake of hearing healthcare and sub-optimal levels of hearing aid use once they are fitted. We chose to address the second problem as this pertains more to the direct clinical consultation, an area with which our research team had more experience. The low level of uptake of hearing healthcare services represents a public health issue which we felt others were better able to address. Estimates of hearing aid non-use amongst adults with acquired hearing loss vary from 5 to 40 % [6–13]. The true extent of non-use is open to debate since different methods of data collection and different lengths of follow-up cloud the picture. We therefore defined the behavioural problem in this context as being the long-term use of hearing aids by adults with acquired hearing loss.

Two systematic reviews summarise a wide range of reported reasons for non-use [26, 27]. Using the COM-B model as a frame of reference highlights that some of the factors reported as influencing hearing aid use relate to capability, e.g. inability to physically manage the hearing aid, forgetting to put the hearing aid in and some to opportunity, e.g. cost, lack of support from family and poor follow-up services and information. Reflective motivation is also a factor, e.g. believing that hearing aid use is stigmatising or assessing hearing aid use as less important than other competing behaviours. Automatic motivational factors have not been explicitly investigated in the context of hearing aid use.

### Step 2—Select the Target Behaviour

**Fig 2:**
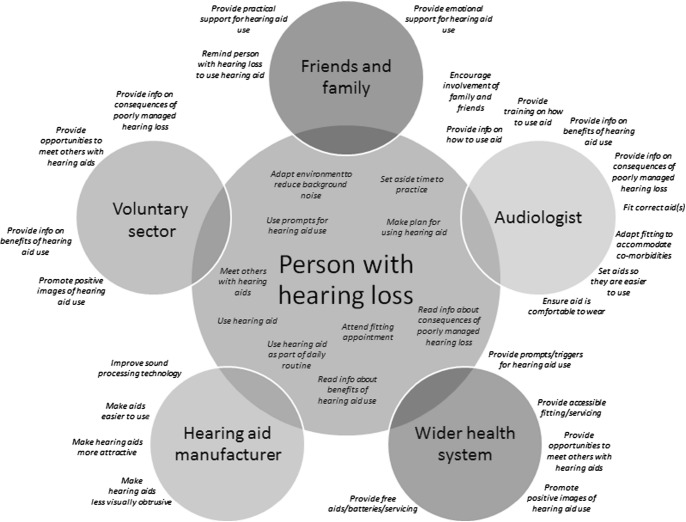
Behaviours relevant to hearing aid use

Using the results of these systematic reviews, supplemented with clinical expertise and psychological theory, we developed a conceptual map of behaviours pertinent to hearing aid use as shown in Fig. 2. Some of these behaviours relate to the person with the hearing loss, but many involve other people, including the audiologists with whom they interact.

Our own systematic review of interventions to improve hearing aid use suggested that some types of SMS can improve some outcomes for adults with acquired hearing loss, at least in the short term. There is a lack of evidence about the effect on long-term outcomes. A lack of evidence and poor description of the active components of interventions also makes it difficult to assess the effect of specific types of SMS on behaviour such as hearing aid use, particularly those that seek to support patients to become involved in their own care [35]. The Delphi review of SMS in adult auditory rehabilitation suggested there is consensus that a range of different types of SMS, including those that seek to actively involve patients, should be a routine part of the clinical consultation [58]. We therefore chose to focus our attention on the interaction between the patient and audiologist and particularly on the fitting consultation since little work have focused on this to date [44].

Figure 2 illustrates the complexity inherent in many behavioural contexts. Clearly, it would be impractical and unnecessary to implement and evaluate an intervention aimed at all these behaviours. For example, some of them may already be addressed by current service provision or research. Our qualitative work with audiologists in hearing aid fittings shows there are opportunities for audiologists to provide information about the benefits of hearing aid use or the consequences of non-use, discuss prompts or cues for hearing aid use and engage in collaborative planning behaviour [59]. These behaviours are not already addressed within current service provision, but evidence suggests they may be effective and are therefore likely to have impact. They will be relatively easy to implement, have the best chance of influencing other behaviours and their delivery will be relatively easy to measure.

To address the behavioural problem of long-term hearing aid use by adults with hearing loss, we therefore chose to develop an intervention targeted at implementing and embedding these audiologist behaviours in the routine hearing aid fitting consultation.

#### Step 3—Specify the Target Behaviour

Our specification of the target behaviours is provided in Table 1.

#### Step 4—Identify What Needs to Change

We used the COM-B model as a framework to analyse what needs to change in order for audiologists to engage in the target behaviours. We have summarised the analysis for each target behaviour below.

##### Provide Information about the Benefits of Hearing Aid Use and the Consequences of Non-Use

The qualitative study of behaviour in routine hearing aid fitting appointments showed that audiologists do provide verbal and written information on some of the health, social and environmental consequences of hearing aid use for patients when they attend for hearing aid fitting. Patients report they might benefit if the content could be supplemented with more information regarding the benefits of hearing aid use and the disbenefits of non-hearing aid use. Delivery of this information would be relatively simple. Audiologists demonstrably already have the physical and psychological capability, social opportunity and reflective and automatic motivation to distribute verbal and written information since our study of behaviour during hearing aid fitting consultations shows they are already doing it. They currently lack physical opportunity in that they do not have access to augmented content that includes a focus on the potential benefits of hearing aid use.

##### Discuss Prompts or Cues for Hearing Aid Use

The provision of a prompt to remind patients to use their hearing aids should also be relatively simple. People with hearing loss attending for hearing aid fitting could be given a prompt as part of the package they already receive and asked to place it somewhere where it will remind them to insert and use their hearing aids, or they could be given instruction on how to decide on and use their own prompt. Deciding what this should be or where the prompt should be placed could form part of a collaborative planning process. Like the provision of information, audiologists lack access to such a cue card for patients. However, this behaviour is less familiar than providing information. The use of prompts and cues in the promotion of habitual behaviour is not common practice in audiology and therefore there is also likely to be an issue with psychological capability in terms of knowing why and how to discuss the use of prompts with patients.

**Table 1:** Specification of target behaviours

Target behaviour	Who	What	When	Where
Provide realistic information of benefits of hearing aid use	Audiologist	Give written info	During each fitting appointment	Fitting room
Provide information on negative consequences on non-use	Audiologist	Give written info	During each fitting appointment	Fitting room
Provide prompts or triggers	Audiologist	Give physical item to act as a cue or discuss other triggers	During each fitting appointment	Fitting room
Collaborate to develop a plan for using aid(s) that promotes habit formation	Audiologist/person with hearing loss	Work together to create a written plan for when, where etc. hearing aid will be used	During each fitting appointment	Fitting room

##### Engage in Collaborative Behaviour Planning

In terms of planning behaviour, audiologists reported psychological capability, physical and social opportunity and reflective and automatic motivation as important drivers for their own behaviour change in hearing aid fitting consultations. The participants reported that they felt they already had the psychological skills and strength to engage in collaborative planning but that it was really important that they gain an understanding of why planning is needed and how to do it.

Participants reported that having access to a planning template would be helpful. It was important that this could be accessed easily from, and attached to, the electronic patient record. However, the biggest factor mediating behaviour in terms of physical opportunity was having time to engage in collaborative planning with patients while meeting requirements to complete other component behaviours important for hearing aid use such as giving instruction and practice at using the aid. The audiologists felt that being part of a team, all of whom were engaged in the same behaviour, would make the behaviour more likely to occur. This influenced motivation but also had practical benefits in terms of the availability of advice.

Participants reported that believing planning to be a good thing was an important motivating factor, mediated by psychological capability. They also felt they would benefit from planning in advance how and where to incorporate collaborative behaviours and planning into their current routines so that it interfered as little as possible with any competing behaviours such as the need to do real ear measurement or give instruction. Participants reported being strongly motivated by seeing a positive outcome for their behaviour either directly or indirectly. They also reported being strongly driven by habitual processes, recognising that they rely on these already to ensure that important parts of the consultation are not forgotten and to reduce mental effort.

### Step 5—Identify Intervention Functions

For each target audiologist behaviour, we used the APEASE criteria to select intervention functions to address the COM-B elements identified in step 4. Psychological capability and physical opportunity were identified as potential drivers relevant to audiologists giving additional information and providing prompts for hearing aid use. The grid published in the BCW guide [52] suggests that education, training, restriction, environmental restructuring and enablement could address these drivers. In this context, restrictive interventions, like formulating rules such that competing information was restricted or reduced, making it more likely that the target information was distributed, were judged to be unacceptable with possible side effects. Providing education and training in how to use prompts to address psychological capability and environmental restructuring to address physical opportunity were judged to meet the APEASE criteria in this context. Enablement was also considered as a possible intervention function but was judged to be unnecessary if the drivers could be addressed using the other three functions.

For the final behaviour of collaborating with patients to create a behavioural plan for hearing aid use, audiologists report that psychological capability, physical and social opportunity and reflective and automatic motivation all play a role. According to the BCW guide, all of the intervention functions might be applicable in this context. Persuasion was judged unlikely to be effective as audiologists did not report that emotion influenced whether they were likely to engage in planning or not. Incentivisation was judged to be impractical in this context. Restriction was judged to be impractical, unacceptable and possibly unsafe. The remaining intervention functions of education, coercion, training, environmental restructuring, modelling and enablement were all judged to be potentially relevant and met the APEASE criteria in this context.

### Step 6—Identify Policy Categories

The potential choice of policy category is more open than when moving from COM-B analysis to intervention function. However, the context within which change is implemented may place more limits on the choice of policy category. This is reflected in the application of the APEASE criteria in the context of this research. We judged communication, fiscal measures and legislation to be either impractical or unlikely to be effective in this context. Environmental planning could be used to deliver the environmental restructuring changes needed so that the working environment of the audiologist is conducive to the behaviour changes to be implemented. This along with changes in service provision specified in the intervention design could deliver all the selected intervention functions and individual behaviour change techniques. Should the intervention prove effective, then the changes in service provision could be written into guidelines, and possibly even a regulatory framework, at a later date. However, at this stage in intervention development and feasibility testing, we judged that environmental restructuring and service provision were appropriate policy categories in this context.

### Step 7—Identify Behaviour Change Techniques

Using the BCW guide [52] and version 1 of the behaviour change technique taxonomy [61], we reviewed the behaviour change techniques (BCTs) most commonly used to deliver each of the selected intervention functions. Using the APEASE criteria once again, we selected appropriate BCTs for each intervention function. A summary of the BCTs employed in this intervention is given in Table 2 alongside the intervention functions that they serve and the COM-B elements that they address. For example, we elected to give information about the health and social consequences of the target behaviours to serve the intervention function of education and address psychological capability (increasing knowledge of why planning is important) and reflective motivation (changing beliefs about the positive value of planning). An element of coercion could be used to address automatic motivation by making it more attractive to engage in planning behaviour than to omit it. Training will be served by a combination of instruction, demonstration and practice at performing the behaviours to influence psychological capability (training in how to make a behaviour plan), physical opportunity (training audiologists how to make plans in a time-efficient way) and automatic motivation (to prompt rehearsal and repetition of planning in a consistent context so that it is more likely to become part of the clinical routine). Providing an example of the target behaviour during training for audiologists to imitate also serves the intervention function of modelling, further addressing automatic motivation. The provision of an accessible template and prompt for planning serves the function of environmental restructuring to address physical opportunity and automatic motivation. Increasing social support from other staff also serves this function by addressing social opportunity. Training that includes an opportunity for audiologists to set goals, action-plan and problem-solve regarding when, where and how they will engage in planning and discuss prompts with their patients during the fitting consultation serves the function of enablement, addressing audiologists’ reflective and automatic motivation.

Since a major portion of this intervention is focused on asking audiologists to help patients make a behaviour plan for hearing aid use, we have called our intervention the ‘I-PLAN’.

### Step 8—Identify Mode of Delivery

Individual BCTs may be delivered in various modes and formats. For example, education may be delivered face-to-face, online or in writing. In this case, since the behaviour that we would like audiologists to try is being conducted face-to-face in fitting consultations, we have chosen to deliver training in a face-to-face format. This will facilitate demonstration and practice of the target behaviours. During the feasibility testing phase of the intervention development and evaluation, the training will be delivered by one of the research team, who is also an audiologist but, in the longer term, the training is designed to be deliverable by any audiologist who has used the intervention with patients. Modelling the intervention they will implement with patients, audiologists will collaborate with a trainer during a 45-min face-to-face group training session to produce an I-PLAN of their own, detailing their behavioural goals, e.g. to create an I-PLAN with their patients, action-planning for how they will do this and problem-solving to address factors that might prevent them from doing it. In creating their ‘plan for planning’ during training, audiologists will use the same electronic template that they will use with patients when planning hearing aid use. The modelling nature of the training is an efficient way to demonstrate both how to make an I-PLAN and the benefits of doing so; both were reported by audiologists as important drivers of behaviour change. The mode of delivery will also reinforce the message that this intervention is time efficient, a component that audiologist participants in the behavioural analysis identified as an important influence on their behaviour in this context. Delivering training that takes longer or is more involved is also felt to be unrealistic in terms of wider, future implementation.

**Table 2:** Active ingredients of the I-PLAN intervention

BCT	Code (from BCTTv1)	Definition (from BCTTv1)	Intervention functions served	COM-B elements addressed
Goal setting (behaviour)	1.1	Set or agree a goal defined in terms of the behaviour to be achieved	Enablement	Auto M Ref M
Problem solving	1.2	Analyse or prompt the person to analyse factors influencing the behaviour and generate or select strategies that include overcoming barriers and/or increasing facilitators	Enablement	Auto M Ref M
Action planning	1.4	Prompt detailed planning of performance of the behaviour (must include one of context, frequency, duration and intensity). Context may be environmental or internal	Enablement	Auto M Ref M
Instruction on how to perform a behaviour	4.1	Advise or agree on how to perform the behaviour	Training	Psych C Phys O AutoM
Information about health consequences	5.1	Provide information (e.g. written, verbal, visual) about health consequences of performing the behaviour	Education	Psych C Auto M Ref M
Information about social and environmental consequences	5.3	Provide information (e.g. written, verbal, visual) about social and environmental consequences of performing the behaviour	Education	Psych C Auto M Ref M
Demonstration of the behaviour	6.1	Provide an observable sample of the performance of the behaviour, directly in person or indirectly e.g. via film, pictures for the person to aspire to or imitate	Training, modelling	Psych C Phys O AutoM
Prompts/cues	7.1	Introduce or define environmental or social stimulus with the purpose of prompting or curing the behaviour. The prompt or cue would normally occur at the time or place of performance	Environmental restructuring	Phys O Auto M
Behavioural practice/ rehearsal	8.1	Prompt practice or rehearsal of the performance of the behaviour one or more times in a context or at a time when the performance may not be necessary, in order to increase habit and skill	Training	Psych C Auto M
Habit formation	8.3	Prompt rehearsal and repetition of the behaviour in the same context repeatedly so that the context elicits the behaviour	Training	Psych C Auto M
Restructuring the social environment	12.2	Change or advise to change the social environment in order to facilitate performance of the wanted behaviour or create barriers to the unwanted behaviour (other than prompts/	Environmental restructuring	Soc O
Adding objects to the environment	12.5	cues, rewards or punishments) Add objects to the environment in order to facilitate performance of the behaviour	Environmental restructuring	Phys O Auto M
Punishment	14.2	Arrange for aversive consequence contingent on the performance of the unwanted behaviour	Coercion	Auto M

During each fitting consultation subsequent to training, audiologists who do not create a patient I-PLAN will be directed, via the electronic patient record, to complete a more complex form outlining the reasons why they did not complete it.

Audiologists will receive an ‘i-can I-PLAN’ card which they will be asked to place somewhere where it will act as a cue to engage in planning for hearing aid use with patients. The reverse of the card lists some evidence-based advantages of such planning for themselves and their patients. Audiologists will also be provided with similar cards to issue to patients who can use them as a prompt to remind them to put their hearing aid in. The reverse of the patient cards will list some potential benefits of hearing aid use. Thus, the educative BCTs of providing information about the consequences of planning and the use of prompts will be provided in written form that mirrors the information provided to patients about the benefits and consequences of hearing aid use.

**Fig 3:**
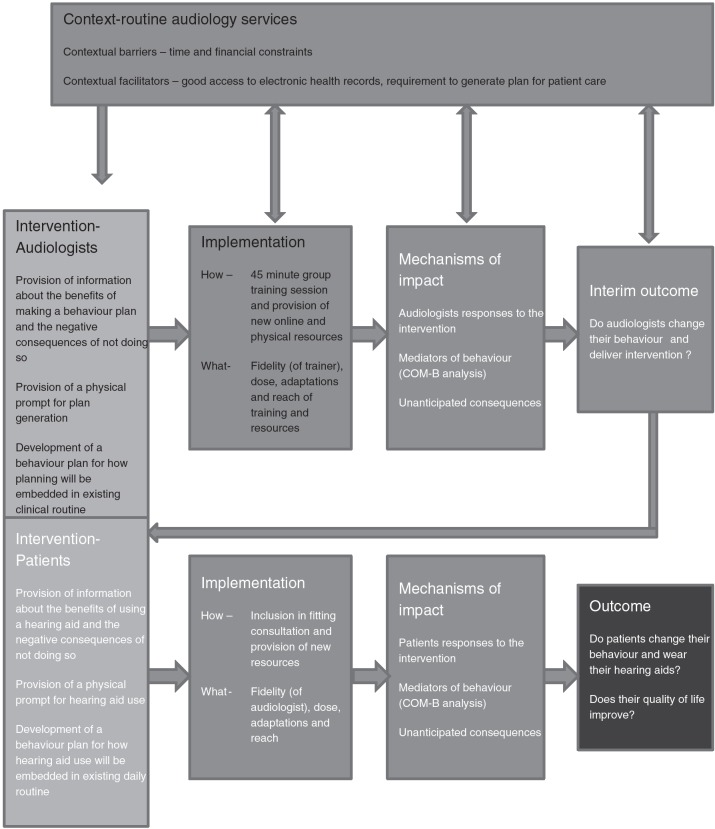
Logic model showing intervention levels and feasibility evaluation as recommended in MRC guidance on process evaluations [64]

Development of individual components of intervention delivery were informed by the active involvement of a steering group consisting of two hearing aid users and two audiologists to ensure that they were acceptable to both sets of stake holders. A logic model for the implementation and evaluation of the intervention is shown in Fig. 3.

## Discussion

This paper describes the development of an intervention to improve hearing aid use in adult auditory rehabilitation, using the behaviour change wheel, to operationalise the development phase of the MRC framework for complex interventions. The intervention is called I-PLAN. To our knowledge, this is the first theory-based intervention to focus on audiologist behaviour during hearing aid fittings.

I-PLAN involves supporting audiologists to provide patients with information about the benefits of hearing aid use and the consequences of non-use, prompts for hearing aid use and a behavioural plan for starting and maintaining hearing aid use. The audiologist-targeted intervention mirrors the elements provided to patients so that they are provided with information about the benefits of their own behaviour change, prompts to carry out the behaviours and a behavioural plan for starting and maintaining the behaviours.

### Comparison with Other Work

The BCW has been used in other contexts to develop interventions. Several illustrative examples are given in the BCW guide, targeting both patient and healthcare professional behaviour [52]. Recently, Sinnott et al. published their description of how they used the BCW to develop an intervention to improve medication management in multi-morbid patients by general practitioners [53]. This detailed description of the development process, using the stages of the BCW, follows the same path as our study. This demonstrates how the same BCW process can be applied in different contexts, producing context-specific theory-based intervention design using a generic process.

### Strengths and Weaknesses

Sinnott et al. give a rounded discussion of the advantages and disadvantages of using a structured, bottom-up approach to intervention development using the BCW [53]. Similarly, we value a systematic and structured approach which allowed the integration of evidence synthesis and generation. Although some subjective judgement is required in operationalising the later steps of the BCW, the structure allows these judgements to be made in a transparent way. Like Sinnott et al., we did find the process to be lengthy, taking 3 years from problem identification to intervention design. However, we did feel that the process might be followed more quickly in other contexts, particularly those where systematic reviews and preparatory relevant qualitative work have already been undertaken.

Unlike Sinnott et al. and other COM-B-based intervention designs [54], we are explicitly addressing two levels of behavioural problem (sub-optimal levels of hearing aid use and sub-optimal engagement in information giving, use of prompts and behavioural plans) in two inter-related target populations (adults with acquired hearing loss and audiologists). This adds an additional level of complexity. A particular strength of the BCW approach is the focus at the outset on understanding the behaviour, the context within which it occurs and the interrelationship between the different people interacting within the system of behaviour. This allowed us to elucidate the links between professional and patient behaviour in this context from the beginning and to incorporate this into the intervention development rather than only during feasibility testing. However, this additional complexity has resulted in a complex, multi-layered intervention. We have also chosen to include several audiologist behaviours rather than focusing on one. We recognise that this will require careful evaluation during the next feasibility testing phase of the research [64].

### Implications for Future Research

This research shows how the BCW can be applied across contexts to address not just a single behavioural problem but sets of inter-related behaviours. The success of this approach however has yet to be tested.

The conceptual map developed as part of this work presents several other promising targets for intervention development in this context, notably for the ongoing work on the involvement of the family and friends of the person with the hearing loss [21, 44, 65–67].

The use of the behaviour change technique taxonomy allows explicit definition of the active ingredients of an intervention, and the link with the BCW means that individual BCTs can be linked back to theory. This should allow replication and facilitate data synthesis. Identification of the active ingredients of an intervention was an issue that arose during our systematic review of interventions to improve hearing aid use [35]. Clearer specification of the links between behaviour and outcome and the techniques employed to change behaviour would be beneficial in future research in hearing healthcare.

## Conclusions

This paper describes the development of an intervention to improve long-term hearing aid use in adult auditory rehabilitation: I-PLAN. The I-PLAN intervention development has followed a systematic, transparent process using the behaviour change wheel to operationalise the first development phase of the MRC framework for complex interventions. This research demonstrates how this approach can be applied in parallel to multi-level behavioural problems. The feasibility of the I-PLAN will be tested in the next phase of this research using a cluster-randomised implementation trial.

## Abbreviations

APEASE: Affordability, practicability, effectiveness/cost-effectiveness, acceptability, side-effects/safety and equity
BCT: Behaviour change technique
BCW: Behaviour change wheel
COM-B: Capability, opportunity, motivation and behaviour model
MRC: Medical Research Council
SMS: Self-management support
WHO: World Health Organisation

## References

[1] World Health Organisation. Chronic conditions: The global burden. Available at http://www.who.int/healthinfo/global_burden_disease/2004_report_update/en/ Accessibility verified July 21, 2016.

[2] World Health Organisation. Deafness and hearing loss. Available at http://www.who.int/mediacentre/factsheets/fs300/en/. Accessibility verified March 7, 2016.

[3] Murray CJ, Richards MA, Newton JN, et al. UK health performance: Findings of the Global Burden of Disease Study. Lancet 2013; 381: 997–1020. doi: 10.1016/S0140-6736(13)60355-4. 10.1016/S0140-6736(13)60355-4 23668584

[4] World Health Organisation. Global health and aging. Available at http://www.who.int/ageing/publications/global_health.pdf. Accessibility verified July 21, 2016.

[5] Pronk M, Kramer SE, Davis AC, et al. Interventions following hearing screening in adults: A systematic descriptive review. Int J Audiol. 2011; 50: 594–609. doi: 10.3109/14992027.2011.582165 21718228 10.3109/14992027.2011.582165

[6] Lupsakko TA, Kautiainen HJ, Sulkava R The non-use of hearing aids in people aged 75 years and over in the city of Kuopio in Finland. Eur Arch Otorhinolaryngol. 2005; 262: 165–169. 15133689 10.1007/s00405-004-0789-x

[7] Gimsing S Use of hearing aids five years after issue. Ugeskr Laeger. 2008; 170: 3407–3411. 18976596

[8] Smeeth L, Fletcher AE, Ng ES, et al. Reduced hearing, ownership, and use of hearing aids in elderly people in the UK—the MRC Trial of the Assessment and Management of Older People in the Community: A cross-sectional survey. Lancet 2002; 359: 1466–1470. 10.1016/s0140-6736(02)08433-7 11988245

[9] Sorri M, Luotonen M, Laitakari K Use and non-use of hearing aids. Br J Audiol. 1984; 18: 169–172. 10.3109/03005368409078944 6487851

[10] Vuorialho A, Sorri M, Nuojua I, Muhli A Changes in hearing aid use over the past 20 years. Eur Arch Otorhinolaryngol. 2006; 263: 355–360. 16283199 10.1007/s00405-005-1007-1

[11] Hartley D, Rochtchina E, Newall P, Golding M, Mitchell P Use of hearing aids and assistive listening devices in an older Australian population. J Am Acad Audiol 2010; 21: 642–653. doi: 10.3766/jaaa.21.10.4. 10.3766/jaaa.21.10.4 21376005

[12] Hougaard S, Ruf S EuroTrak I: A consumer survey about hearing aids in Germany, France, and the UK. Hearing Review 2011; 18: 12–28.

[13] Kochkin S MarkeTrak V “Why my hearing aids are in the drawer: The consumers’ perspective. Hearing Journal. 2000; 53: 34–42.

[14] Arlinger S Negative consequences of uncorrected hearing loss-a review. Int J Audiol. 2003; 42 Suppl 2: 17–20. 12918624

[15] Brooks D, Hallam R, Mellor P The effects on significant others of providing a hearing aid to the hearing-impaired partner. Brit J Audiol. 2001; 35: 165–171. 11548043 10.1080/00305364.2001.11745234

[16] Hallberg LRM, Barrenäs ML. Living with a male with noise-induced hearing loss: Experiences from the perspective of spouses. Brit J Audiol. 1993; 27: 255–261. 8312848 10.3109/03005369309076702

[17] Hickson L, Worrall L Beyond hearing aid fitting: Improving communication for older adults. Int J Audiol. 2003; 42 Suppl 2: 84–91. 12918634

[18] Chia EM, Wang JJ, Rochtchina E, Cumming RR, Newall P, Mitchell P Hearing impairment and health-related quality of life: The Blue Mountains Hearing Study. Ear Hear. 2007; 28: 187–195. 17496670 10.1097/AUD.0b013e31803126b6

[19] Heine C, Browning CJ The communication and psychosocial perceptions of older adults with sensory loss: A qualitative study. Ageing Soc. 2004; 24: 113–130.

[20] Mulrow CD, Aguilar C, Endicott JE, Velez R, Tuley MR, Charlip WS, Hill JA Association between hearing impairment and the quality of life of elderly individuals. J Am Geriatr Soc. 1990; 38: 45–50. 2295767 10.1111/j.1532-5415.1990.tb01595.x

[21] Stark P, Hickson L Outcomes of hearing aid fitting for older people with hearing impairment and their significant others. Int J Audiol. 2004; 43: 390–398. 15515638 10.1080/14992020400050050

[22] Boi R, Racca L, Cavallero A, et al. Hearing loss and depressive symptoms in elderly patients. Geriatr Gerontol Int 2012; 12: 440–445. doi: 10.1111/j.1447-0594.2011.00789.x. 10.1111/j.1447-0594.2011.00789.x 22212622

[23] Gopinath B, Hickson L, Schneider J, et al. Hearing-impaired adults are at increased risk of experiencing emotional distress and social engagement restrictions five years later. Age Ageing 2012; 41: 618–623. doi: 10.1093/ageing/afs058. 10.1093/ageing/afs058 22591986

[24] Saito H, Nishiwaki Y, Michikawa T, et al. Hearing handicap predicts the development of depressive symptoms after 3 years in older community-dwelling Japanese. J Am Geriatr Soc 2010; 58: 93–97. doi: 10.1111/j.1532-5415.2009.02615.x. 10.1111/j.1532-5415.2009.02615.x 20002512

[25] Lin FR Hearing loss and cognition among older adults in the United States. J Gerontol A Biol Sci Med Sci. 2011; 66: 1131–1316. doi: 10.1093/gerona/glr115. 21768501 10.1093/gerona/glr115 PMC3172566

[26] McCormack A, Fortnum H Why do people fitted with hearing aids not wear them? Int J Audiol. 2013; 52: 360–368. doi: 10.3109 /14992027.2013.769066. 23473329 10.3109/14992027.2013.769066 PMC3665209

[27] Ng JH, Loke AY Determinants of hearing-aid adoption and use among the elderly: A systematic review. Int J Audiol. 2015; 54: 291–300. doi: 10.3109/14992027.2014.966922. 25640403 10.3109/14992027.2014.966922

[28] Dunear-Jacob J, Erlen JA, Schlenk EA, Ryan CM, Sereika SM Adherence in chronic disease. Annu Rev Nurs Res 2000; 18: 48–90. 10918932

[29] Haskard KB, DiMatteo MR, Williams SL Adherence and health outcomes: How much does adherence matter? In: Shumaker SA, Ockene JK, Riekert KA, eds. The handbook of health behaviour change. 3rd ed. New York: Springer; 2009: 771–784.

[30] Adams K, Greiner, AC, Corrigan, JM. 1st Annual Crossing the Quality Chasm Summit: A focus on communities. Washington: National Academies Press; 2004. 25009886

[31] Bodenheimer T, Wagner EH, Grumbach K Improving primary care for patients with chronic illness. JAMA 2002; 288: 1775–1779. 10.1001/jama.288.14.1775 12365965

[32] Boger E, Ellis J, Latter S, et al. Self-management and self-management support outcomes: A systematic review and mixed research synthesis of stakeholder views. PloS one 2015; 10:e0130990. doi: 10.1371/journal.pone.0130990. 10.1371/journal.pone.0130990 PMC4498685 26162086

[33] Lorig KR, Holman HR Self-management education: History, definition, outcomes, and mechanisms. Ann Behav Med. 2003; 26: 1–7. 12867348 10.1207/S15324796ABM2601_01

[34] Pearson ML, Mattke S, Shaw R, Ridgely, MS, Wiseman, SH. Patient Self-Management Support Programs.Rockville: Agency for Healthcare Research and Quality; 2007.

[35] Barker F, Mackenzie E, Elliott L, Jones S de Lusignan S Interventions to improve hearing aid use in adult auditory rehabilitation. Cochrane Database of Systematic Reviews. 2014; doi: 10.1002/14651858. 10.1002/14651858.CD010342.pub2 25019297

[36] Hibbard JH, Mahoney ER, Stock R, Tusler M Do Increases in Patient Activation Result in Improved Self-Management Behaviors? Health Serv Res. 2007; 42: 1443–1463. 17610432 10.1111/j.1475-6773.2006.00669.x PMC1955271

[37] Mead N, Bower P Patient-centredness: A conceptual framework and review of the empirical literature. Soc Sci Med 2000; 51: 1087–1110. 10.1016/s0277-9536(00)00098-8 11005395

[38] Barker F, MacKenzie E, Elliott L, de Lusignan S Outcome Measurement in Adult Auditory Rehabilitation: A Scoping Review of Measures Used in Randomized Controlled Trials. Ear Hear. 2015; 36: 567–573. doi: 10.1097/AUD.0000000000000167. 25919402 10.1097/AUD.0000000000000167

[39] Hanratty B, Lawlor DA Effective management of the elderly hearing impaired—a review. J Pub Health. 2000; 22: 512–517. 10.1093/pubmed/22.4.512 11192279

[40] Grenness C, Hickson L, Laplante-Levesque A, Davidson B Patient-centred audiological rehabilitation: Perspectives of older adults who own hearing aids. Int J Audiol. 2014; 53 Suppl 1: 68–75. doi: 10.3109/14992027.2013.866280. 10.3109/14992027.2013.866280 24528290

[41] Grenness C, Hickson L, Laplante-Levesque A, Meyer C, Davidson B The nature of communication throughout diagnosis and management planning in initial audiologic rehabilitation consultations. J Am Acad Audiol. 2015; 26: 36–50. doi: 10.3766/jaaa.26.1.5. 25597459 10.3766/jaaa.26.1.5

[42] Kelly TB, Tolson D, Day T, McColgan G, Kroll T, Maclaren W Older people’s views on what they need to successfully adjust to life with a hearing aid. Health Soc Care Community. 2013; 21: 293–302. doi: 10.1111/hsc.12016. 23373520 10.1111/hsc.12016 PMC3712468

[43] Laplante-Levesque A, Knudsen LV, Preminger JE, et al. Hearing help-seeking and rehabilitation: Perspectives of adults with hearing impairment. Int J Audiol. 2012; 51: 93–102. doi: 10.3109 /14992027.2011.606284. 21942678 10.3109/14992027.2011.606284

[44] Knudsen LV, Oberg M, Nielsen C, Naylor G, Kramer SE Factors influencing help seeking, hearing aid uptake, hearing aid use and satisfaction with hearing aids: A review of the literature. Trends Amplif. 2010; 14: 127–154. doi: 10.1177/1084713810385712. 21109549 10.1177/1084713810385712 PMC4111466

[45] Campbell M, Fitzpatrick R, Haines A, et al. Framework for design and evaluation of complex interventions to improve health .BMJ. 2000; 321: 694–696. 10.1136/bmj.321.7262.694 PMC1118564 10987780

[46] Craig P, Dieppe P, Macintyre S, Michie S, Nazareth I, Petticrew M Developing and evaluating complex interventions: The new Medical Research Council guidance. BMJ. 2008; 337: 1655. doi: 10.1136/bmj.a1655. 10.1136/bmj.a1655 PMC2769032 18824488

[47] Kleinman LC, Dougherty D Assessing quality improvement in health care: theory for practice. Pediatrics. 2013; 131 Suppl 1: 110–119. doi: 10.1542/peds.2012-1427n. 10.1542/peds.2012-1427n 23457146

[48] Marcus BH, Williams DM, Dubbert PM, et al. Physical Activity Intervention Studies What We Know and What We Need to Know: A Scientific Statement From the American Heart Association Council on Nutrition, Physical Activity, and Metabolism (Subcommittee on Physical Activity); Council on Cardiovascular Disease in the Young; and the Interdisciplinary Working Group on Quality of Care and Outcomes Research. Circulation. 2006; 114: 2739–2752. 10.1161/CIRCULATIONAHA.106.179683 17145995

[49] Michie S, Johnston M, Abraham C, Lawton R, Parker D, Walker A Making psychological theory useful for implementing evidence based practice: A consensus approach. Qual Saf Health Care. 2005; 14: 26–33. 10.1136/qshc.2004.011155 PMC1743963 15692000

[50] Eccles M, Grimshaw J, Walker A, Johnston M, Pitts N Changing the behavior of healthcare professionals: The use of theory in promoting the uptake of research findings. J Clin Epidemiol 2005; 58: 107–112. 10.1016/j.jclinepi.2004.09.002 15680740

[51] Michie S, van Stralen MM, West R The behaviour change wheel: A new method for characterising and designing behaviour change interventions. Implement Sci 2011; 6: 42. doi: 10.1186/1748-5908-6-42. 21513547 10.1186/1748-5908-6-42 PMC3096582

[52] Michie S, Atkins L, West R The behaviour change wheel: A guide to designing interventions. London: Silverback; 2014.

[53] Sinnott C, Mercer SW, Payne RA, Duerden M, Bradley CP, Byrne M Improving medication management in multimorbidity: Development of the MultimorbiditY COllaborative Medication Review And DEcision Making (MY COMRADE) intervention using the Behaviour Change Wheel. Implement Sci 2015; 10: 132. doi: 10.1186/s13012-015-0322-1. 10.1186/s13012-015-0322-1 PMC4582886 26404642

[54] Alexander KE, Brijnath B, Mazza D Barriers and enablers to delivery of the Healthy Kids Check: An analysis informed by the Theoretical Domains Framework and COM-B model. Implement Sci 2014; 9: 60. doi: 10.1186/1748-5908-9-60. 10.1186/1748-5908-9-60 PMC4047437 24886520

[55] Jackson C, Eliasson L, Barber N, Weinman J Applying COM-B to medication adherence. European Health Psychologist 2014; 16: 7–17.

[56] Moodie ST Spotlight on Science: Implementation Science. Canadian Hearing Report. 2011; 6: 23–26.

[57] Humes LE, Krull V Hearing aids for adults. In Wong L, Hickson L, eds. Evidence-based practice in audiology. San Diego, CA: Plural; 2012: 61–91

[58] Barker F, Munro KJ, de Lusignan S Supporting living well with hearing loss: A Delphi review of self-management support. Int J Audiol 2015; 54: 691–699 10.3109/14992027.2015.1037019 25938504

[59] Barker F, Mackenzie E, de Lusignan S Current process in hearing aid fitting appointments: an analysis of audiologists’ use of behaviour change techniques using the behaviour change technique taxonomy (v1). Int J Audiol 2016; doi:10.1080/14992027.2016.1197425. 10.1080/14992027.2016.1197425 27366971

[60] Barker F, Atkins L, de Lusignan S Applying the COM-B behaviour model and behaviour change wheel to develop an intervention to improve hearing-aid use in adult auditory rehabilitation. Int J Audiol 2016; doi:10.3109/14992027.2015.1120894. 10.3109/14992027.2015.1120894 27420547

[61] Michie S, Richardson M, Johnston M, et al. The behavior change technique taxonomy (v1) of 93 hierarchically clustered techniques: Building an international consensus for the reporting of behavior change interventions. Ann Behav Med 2013; 46: 81–95. doi: 10.1007/s12160-013-9486-6. 10.1007/s12160-013-9486-6 23512568

[62] Chisolm TH, Johnson CE, Danhauer JL, et al. A systematic review of health-related quality of life and hearing aids: Final report of the American Academy of Audiology Task Force On the Health-Related Quality of Life Benefits of Amplification in Adults. J Am Acad Audiol 2007; 18: 151–183. 10.3766/jaaa.18.2.7 17402301

[63] Chisolm TH, Arnold M Evidence about the effectiveness of aural rehabilitation programs for adults. In: Wong L, Hickson L, eds. Evidence-based practice in audiology. San Diego, CA: Plural; 2012: 237–266.

[64] Moore GF, Audrey S, Barker M, et al. Process evaluations of complex interventions: UK Medical Research Council guidance. BMJ 2015; 350: h1258. 10.1136/bmj.h1258 PMC4366184 25791983

[65] Kramer SE, Allessie GH, Dondorp AW, Zekveld AA, Kapteyn TS A home education program for older adults with hearing impairment and their significant others: A randomized trial evaluating short- and long-term effects. Int J Audiol 2005; 44: 255–264. 10.1080/14992020500060453 16028788

[66] Knudsen LV, Laplante-Levesque A, Jones L, et al. Conducting qualitative research in audiology: A tutorial. Int J Audiol 2012; 51: 83–92. doi: 10.3109/14992027.2011.606283. 10.3109/14992027.2011.606283 21916797

[67] Ekberg K, Meyer C, Scarinci N, Grenness C, Hickson L Family member involvement in audiology appointments with older people with hearing impairment. Int J Audiol 2015; 54: 70–76. doi: 10.3109/14992027.2014.948218. 25141941 10.3109/14992027.2014.948218

